# Supplementary material for: Asynchronous magnetic resonance elastography: Shear wave speed reconstruction using noise correlation of incoherent waves
Source: Magn Reson Med. 2022 Oct 27;89(3):990–1001. doi: 10.1002/mrm.29502 (PMC9792433; doi:10.1002/mrm.29502)
Supplement: Supplementary file 1 — DATA S1 MATLAB code used to generate the numerical simulations of Figure 1 [file MRM-89-990-s002.zip › k-Wave/helpfiles/k-wave_initial_value_problems.html]

Initial Value Problems (k-Wave)


# Initial Value Problems

- Example: Homogenous Propagation Medium
- Example: Using A Binary Sensor Mask
- Example: Defining A Sensor Mask By Opposing Corners
- Example: Loading External Image Maps
- Example: Heterogeneous Propagation Medium
- Example: Saving Movie Files
- Example: Recording The Particle Velocity
- Example: Defining A Gaussian Sensor Frequency Response
- Example: Comparison Of Modelling Functions
- Example: Setting An Initial Pressure Gradient
- Example: Simulations In One Dimension
- Example: Simulations In Three Dimensions
- Example: Simulations In An Axisymmetric Coordinate System
- Example: Photoacoustic Waveforms in 1D, 2D and 3D
